# The Emerging Melanoma Management: Historical Perspective to Future Directions

**DOI:** 10.3390/cancers18060968

**Published:** 2026-03-17

**Authors:** Shin Yee Hui, Rohit Jain, Nikolas K. Haass, Wolfgang Weninger, Shweta Tikoo, Dajiang Guo

**Affiliations:** 1 Department of Anatomical and Cellular Pathology and State Key Laboratory of Translational Oncology, Prince of Wales Hospital, The Chinese University of Hong Kong, Hong Kong, China; shinyeehui@cuhk.edu.hk; 2 Li Ka Shing Institute of Health Sciences, Faculty of Medicine, The Chinese University of Hong Kong, Hong Kong, China; 3 Department of Dermatology, Medical University of Vienna, 1090 Vienna, Austria; rohit.jain@meduniwien.ac.at (R.J.); w.weninger@meduniwien.ac.at (W.W.); 4 Frazer Institute, The University of Queensland, Brisbane, QLD 4072, Australia; n.haass1@uq.edu.au

**Keywords:** melanoma, skin cancer, targeted therapy, immunotherapy

## Abstract

Cutaneous melanoma is an aggressive form of cancer that accounts for approximately 80% of skin cancer-associated mortality. Treatment strategies for melanoma have been rapidly evolving over the past two decades. Clinical outcomes for advanced melanoma patients have improved with the use of immune checkpoint inhibitors and targeted therapies, including combination therapies. In this review, we primarily focus on the evolution of novel therapies for advanced melanoma patients, existing and emerging melanoma biomarkers, and the most up-to-date treatment recommendations. Furthermore, we summarise ongoing studies, clinical trials, and emerging therapeutic strategies. In this review, we will walk you through how cutaneous melanoma treatment strategies have evolved over the past 50 years. We will provide background on how those strategies were developed, how they function, their pros and cons, and their performance in terms of clinical benefits. Lastly, we will provide insights into potential therapies and research areas. In summary, improving the efficacy of immune checkpoint blockade remains the most promising approach for improving cutaneous melanoma management. However, strategies such as CAR-T/NK, PROTAC, or extracellular vesicle-based therapies are also worth further investigation.

## Simple Summary

Cutaneous melanoma accounts for the majority of skin cancer-caused deaths. Melanoma management plans have been evolving rapidly. Here, we summarise clinical biomarkers and approved treatment options for cutaneous melanoma to date. We have compared the pros and cons of the latest therapies in terms of clinical benefits and discussed future directions for emerging therapeutic strategies. In summary, immune checkpoint blockade therapy remains the main focus of current studies; new combinations of approved immune checkpoint blockades or the addition of novel immune checkpoint inhibitors are expected to continue improving clinical outcomes, although CAR-T/CAR-NK, PROTAC-based targeted therapies, and extracellular vesicle-based treatment options show significant potential.

## 1. Introduction

Melanoma arises from the malignant transformation of melanocytes, which are neural crest-derived, pigment-producing cells. Melanoma is one of the most invasive skin cancers with a significantly high risk of mortality [1]. Despite contributing to <5% of all skin cancers, melanoma accounts for approximately 80% of skin cancer-associated deaths [2,3]. The most common form of melanoma is cutaneous melanoma [4]; however, rarer forms such as mucosal melanoma [5], acral melanoma [5], and uveal melanoma [6] are generally associated with poorer patient outcomes [7,8,9]. The last two decades have seen vast shifts in the treatment landscape of advanced cutaneous melanoma, leading to remarkable improvements in patient prognosis. However, disparities in treatment outcomes exist among individual patients; therefore, intensive efforts are underway to expand these therapeutic gains to a broader repertoire of patients. In this review, we aim to provide an overview of the evolution of cutaneous melanoma management and discuss future directions for novel therapeutic strategies.

## 2. Melanoma Mutation Load and Treatments

Melanoma exhibits one of the highest mutational burdens among all human cancers [10]. The most commonly reported driver mutations result in aberrant activation of the mitogen-activated protein kinase (MAPK) pathway or the PI3-Kinase pathway, leading to enhanced cell proliferation, differentiation, and survival [10,11]. The most frequently altered genes include *BRAF*, *NRAS*, *NF1*, *CDKN2A*, phosphatase and tensin homologue (*PTEN*), KIT proto-oncogene (*KIT*), tumour protein P53 (*TP53*), and telomerase reverse transcriptase (*TERT*) [10,12]. BRAF is a serine/threonine protein kinase in the MAPK pathway. BRAF-V600 mutation is the most common driver mutation in melanoma, affecting approximately 50% of all cutaneous melanoma patients [4]. Among these cases, most harbour the BRAF-V600E mutations. BRAF-V600E involves the substitution of valine (V) by glutamic acid (E), resulting in the constitutively active conformation of the catalytic domain of the BRAF protein, and a 500-fold higher kinase activity compared to the wild-type BRAF kinase [13]. A total of 10–30% of these cases may harbour BRAF-V600K mutation, which involves the substitution of valine (V) by lysine (K) in BRAF [14]. Melanoma patients with disseminated metastasis are typically tested for BRAF-V600 mutations. Those with a favourable BRAF-V600 mutation profile can undergo BRAF-targeted therapy (Vemurafenib, Encorafenib, and Dabrafenib). BRAF inhibitors aim to inhibit the aberrant MAPK pathway activation by targeting the BRAF mutations, thereby inducing melanoma cell death and tumour regression [15]. BRAF inhibitors are always used in combination with MEK inhibitors such as Binimetinib, Cobimetinib, and Trametinib [16,17,18,19]. The presently approved BRAF and MEK inhibitor combinations include dabrafenib plus trametinib, encorafenib plus binimetinib, and vemurafenib plus cobimetinib [20].

To date, surgery remains the gold standard of care for localised cutaneous melanoma. While surgical excision is an effective management strategy for patients with early-stage melanoma, late-stage melanomas remain notably challenging to treat due to their refractory nature [21,22]. Fortunately, melanoma treatment has undergone a revolution with the emergence of tyrosine kinase inhibitors and immune checkpoint inhibitors. Anti-PD-1 (Programmed cell death protein 1/Programmed death-ligand 1) and anti-CTLA-4 (cytotoxic T-lymphocyte-associated protein 4) therapies have especially achieved outstanding clinical outcomes [23,24]. However, their long-term clinical benefits may be restricted due to issues such as drug resistance and toxicity [15,23,24]. With the introduction of novel immunotherapies, the five-year survival rate for patients with unresectable stage III or stage IV melanoma has improved to approximately 52% [25]. Interestingly, a combination of molecular-targeted therapy and immunotherapy has been poised to yield better results in patients with resected advanced melanoma [22,26,27]. Yet, the only FDA-approved (2020) combined targeted and immunotherapy is Atezolizumab (anti-PD-L1)-Cobimetinib-Vemurafenib for unresectable or metastatic melanoma bearing the BRAF V600 mutation. These developments suggest that joint efforts should be made to explore novel targets for both immunotherapy and targeted therapy. To this end, novel cellular models and drug screening platforms have been developed, and multiple potential targeted therapies have been identified [28,29]. For example, potent inhibitors of ATM kinase have been indicated to suppress melanoma progression and metastasis [29].

## 3. Timeline of Therapeutic Strategies Targeting Cutaneous Melanoma

**Figure 1:**
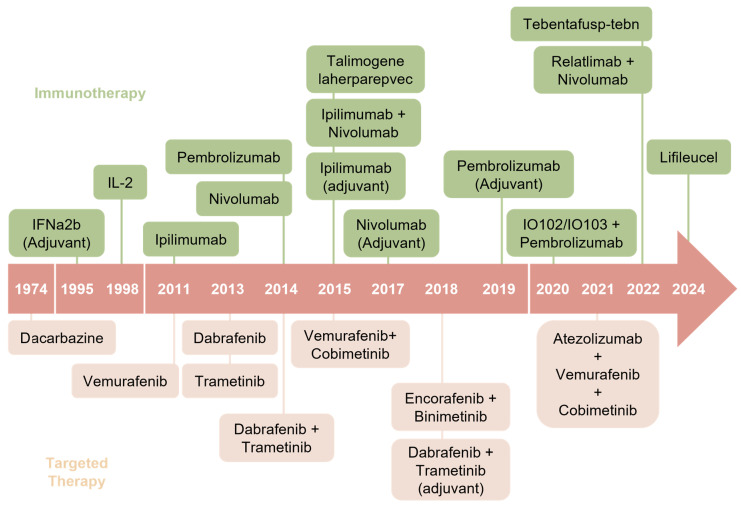
Timeline of novel therapies for cutaneous melanoma. Schematic highlighting the timeline of FDA-approved therapies for advanced melanoma patients.

As previously mentioned, a significant increase in novel melanoma therapies has been observed over the past two decades (Figure 1), leading to substantial improvements in the prognosis of patients with advanced melanoma [22]. However, before BRAF-mutant inhibitors were approved by the FDA in 2011, treatment options for advanced melanoma were limited. In the following section, we aim to present a timeline illustrating the evolution of various melanoma treatment strategies.

### 3.1. DTIC

The first chemotherapeutic agent for metastatic melanoma, dacarbazine (DTIC), was approved in 1974. It was postulated that dacarbazine acts as an alkylating agent by disrupting de novo purine synthesis [30]. The clinical trials of dacarbazine were initiated after Shealy et al. found that it significantly increased the lifespan of mice bearing leukaemia L1210 [31]. However, the long-term, sustained complete response was observed in fewer than 5% of melanoma patients who underwent dacarbazine treatment, with no significant improvement in overall survival [32,33]. A meta-analysis by Lui et al. summarised the low response rate in patients receiving dacarbazine monotherapy (n = 1390), ranging from 5.3% to 28%. The authors also found no significant clinical benefit in dacarbazine combination treatments, thus concluding that dacarbazine generally yields poor outcomes [34].

### 3.2. Interferon a-, IL-2, Ontak

In the 1990s, several immunotherapies, including Interferon-alpha-2b, interleukin-2 (IL-2), and the Treg inhibitor Ontak (Denileukin diftitox), were introduced [1]. The approvals for interferon alpha-2b and recombinant human IL-2 (Aldesleukin) were granted in 1995 and 1998, respectively. A pioneering study from the Eastern Cooperative Oncology Group (ECOG) demonstrated a prolongation of relapse-free survival (RFS) (1 vs. 1.7 years) and overall survival (OS) (2.8 vs. 3.8 years) in melanoma patients who had received interferon alpha-2b as adjuvant therapy following surgery [35]. However, its high toxicity and modest clinical benefits limited its use as a single agent. It was substituted with anti-PD-1 (Pembrolizumab/Nivolumab), which demonstrated a better safety profile and improved RFS [36,37].

IL-2 is a cytokine that binds to trimeric IL-2 receptors (IL-2R) expressed on immunosuppressive regulatory T (Treg) cells at low doses. At higherdoses, IL-2 binds to dimeric IL-2R expressed on effector T (Teff) and natural killer (NK) cells [38]. The stimulatory activity of IL-2 prompted investigations into high-dose human IL-2 for the treatment of metastatic melanoma [38]. Ontak is a diphtheria toxin-based recombinant fusion protein that, when internalised by IL-2 receptor (CD25)-bearing Treg cells, results in cell apoptosis [39,40,41]. The development of Ontak was based on the understanding that the regulatory T cells (Tregs) play a role in suppressing anti-tumour immunity [42]. A study by Jones et al. demonstrated that eliminating Tregs not only suppresses melanoma growth but also induces protective immunity that resists subsequent tumour challenges [43]. Interestingly, the group demonstrated that depletion of Tregs in the murine colorectal tumour CT26 induces tumour immunity not restricted to colorectal cancer, but also extending to B cell lymphoma and renal cell carcinoma [44]. Ontak has consistently demonstrated impressive clinical benefits in patients with cutaneous T cell lymphoma (CTCL) [39,40]. However, its efficacy in clinical melanoma has been inconsistent. In 2005, Attia et al. reported that Ontak failed to deplete Tregs and showed no objective response in metastatic melanoma [45]. In contrast, Telang and colleagues reported that Ontak can transiently deplete Tregs, accompanied by tumour regression in melanoma patients [46,47]. To date, the clinical trials in melanoma have not progressed beyond phase II, rendering the clinical efficacy of Ontak uncertain. Ontak was discontinued in 2014 due to manufacturing issues. A more bioactive and pure version, E7777, has since been developed and is currently being evaluated in clinical trials [48].

### 3.3. BRAF Inhibitors—Vemurafenib/Dabrafenib

In 2002, large-scale sequencing of multiple cancer cell lines revealed a high frequency (66%) of oncogenic *BRAF* somatic mutations in melanoma, most of which harbour an activating missense mutation (V600E), making BRAF an attractive target for treatment [13]. However, it was not until 2008 that the BRAF kinase inhibitor, Vemurafenib (PLX4032), was developed [49,50]. Vemurafenib exhibits potent anticancer activity against melanoma cells with a BRAF V600E mutation, while sparing cells expressing the wild-type BRAF [49,50]. In clinical trials, Vemurafenib demonstrated remarkable efficacy and was approved by the FDA in 2011 for the treatment of *BRAF*-mutant melanoma. A phase II trial reported an overall response rate of 53% in patients with *BRAF*-mutant melanoma who had received first-line therapy [51]. A subsequent phase III trial confirmed that Vemurafenib improved the overall survival rate of melanoma patients with the BRAF V600E mutation compared to the dacarbazine group (84% vs. 64%) [52].

Similar to Vemurafenib, Dabrafenib targets mutant BRAF kinase, which constitutively activates the MAPK signalling pathway. Despite their comparable clinical efficacy, Vemurafenib but not Dabrafenib reduced the frequency, phenotype, and function of circulating CD4^+^ T cells in melanoma patients. Given these unexpected findings, it was suggested that Vemurafenib may hamper anti-tumour immunity in melanoma [53]. Additionally, Vemurafenib has been more frequently associated with nephrotoxicity due to ferrochelatase inhibition in renal tubular epithelial cells [54,55]. Dabrafenib later gained regulatory approval as a single agent for the treatment of *BRAF*-mutant melanoma in 2013 [56,57]. Despite the initial success of BRAF inhibitors, melanoma patients often acquire treatment resistance through activation of alternative pathways, e.g., MAPK and PI3K/Akt signalling [47]. To address these limitations, novel compound screening, e.g., Indolium 1 [58], and combining BRAF inhibitors with immunotherapy have been proposed.

### 3.4. CTLA4 Inhibitor

In addition to targeted therapies such as Vemurafenib, the FDA approved Ipilimumab (developed by Bristol Myers Squibb) for the treatment of advanced melanoma in 2011. Ipilimumab is a humanised monoclonal antibody that inhibits the binding of cytotoxic T-lymphocyte-associated antigen-4 (CTLA-4) to its ligands, CD80 and CD86, which are found on antigen-presenting cells [24]. The discovery of CTLA-4 as an immune checkpoint molecule is widely credited to the work of James P Allison, who was later honoured with the Nobel Prize in Physiology and Medicine [59]. In the 1990s, Allison and colleagues observed that CTLA-4 serves as a brake on T cells, suppressing anti-tumour activity [60]. The group generated anti-CTLA-4 antibodies and found that blocking CTLA-4 unleashed the T cell cytotoxic activity and resulted in tumour rejection in vivo [60,61].

A phase II trial of Ipilimumab as a single agent in pretreated advanced melanomas showed a dose-dependent overall response rate (0% for 0.3 mg/kg, 4.2% for 3 mg/kg, 11% for 10 mg/kg; *p* = 0.002) [62]. In chemotherapy-naïve melanomas, a 5.4% objective response rate was observed with Ipilimumab alone at 3 mg/kg. In contrast, the combined Ipilimumab–dacarbazine group achieved a 17.1% response rate with manageable adverse events in both groups [63]. These findings prompted a subsequent Phase III clinical evaluation of Ipilimumab (10 mg/kg) plus Dacarbazine (850 mg per square metre) as a first-line treatment in metastatic melanoma. In this study, patients receiving combination therapy showed significantly improved OS (median duration of best overall response, 19.3 months) compared with the dacarbazine group (8.1 months) [64]. In addition to the combined chemotherapeutic strategies, the combination with a cancer vaccine, such as the glycoprotein 100 (gp100) peptide vaccine, was also explored. However, it did not enhance clinical outcomes. In a pivotal Phase III trial, Ipilimumab alone showed improved median overall survival (MOS) in metastatic melanoma (10.0 months) compared to the Ipilimumab-gp100 vaccine, or the gp100 vaccine alone, which were 10.1 months and 6.4 months, respectively [24]. However, one obvious disadvantage of anti-CTLA-4 therapy is that it could easily trigger immune-related adverse events (irAEs). Therefore, despite prolonged survival, Ipilimumab therapy is frequently associated with irAEs [65,66].

### 3.5. MEK Inhibitors

Preclinical work highlighted the importance of MEK inhibition [67,68], which was then followed by clinical trials [69,70,71]. Following the approval of the CTLA-4 inhibitor, the FDA approved the MEK inhibitor, Trametinib, in 2013 as a monotherapy for the treatment of *BRAF*-mutant melanoma. Trametinib is a potent allosteric inhibitor of MEK1 and MEK2, which phosphorylate and activate the MAPK/ERK signalling cascade [72]. Although *MEK* itself is not an oncogene, its in vitro activation has been shown to transform cells to become tumorigenic [73]. Since MEK functions downstream of BRAF in the MAPK pathway and has been implicated in the progression of various human cancers, it represents a rational molecular target [74,75].

Although Trametinib was approved for *BRAF*-mutant melanoma, an earlier study demonstrated its antitumour activity in both *BRAF*-wild-type and BRAF-mutant melanoma, with response rates of 33% and 10%, respectively. The data not only highlighted the broader antitumour activity of Trametinib across different melanoma subgroups but also a better safety profile compared to selective BRAF inhibitors [76]. Shortly thereafter, the FDA approved the Dabrafenib–Trametinib combination for the treatment of advanced melanoma. Since patients receiving either a BRAF or a MEK inhibitor alone developed resistance quickly, the data indicated that more complete MAPK pathway inhibition might be required. A few mechanisms were postulated to reactivate MAPK pathway and result in treatment resistance, including (1) upstream activating mutations, (2) downstream MAPK pathway alterations, (3) activation of alternative signalling, and (4) BRAF amplification or alternative splicing [77].

Approval of the Dabrafenib–Trametinib combination was based on a randomised Phase III trial reported by Flaherty et al. The investigators demonstrated that a combination of Dabrafenib and Trametinib substantially prolonged patients’ median duration of response compared with dabrafenib monotherapy (10.5 months vs. 5.6 months) and concluded that such a combination could delay BRAF-inhibitor-acquired resistance [78]. In resected BRAFV600-mutant, stage III melanoma, adjuvant dabrafenib–trametinib also significantly reduced the risk of relapse [26]. However, recent guidelines suggest combination nivolumab–ipilimumab as the preferred first-line therapy over BRAF/MEK inhibitor combination therapy for unresectable or metastatic BRAFV600-mutant melanoma [79]. This recommendation was made because nivolumab–ipilimumab shows more durable survival benefits, regardless of *BRAF* mutation status, compared to the Dabrafenib–Trametinib regimen (5-year OS of 60% vs. 34%, MOS >60.0 months vs. 25.9 months) [25,80].

### 3.6. PD-1/PD-L1 Inhibitors and Talimogene Laherparepvec

Similar to CTLA-4, PD-1 is a co-inhibitory receptor that negatively regulates T cell clonal expansion [81]. PD-1 was discovered a few years after CTLA-4 by the research team led by a Japanese physician–scientist, Tasuku Honjo [82]. Later work by Dr. Minato with Dr. Honjo revealed the potential of blocking the PD-1/PD-L1 axis as a promising cancer therapeutic strategy. The group found that administration of antibodies against PD-l ligand (PD-L1) drastically suppressed tumour growth, accompanied by restored CD8^+^ T cell function in vivo [83]. In 2014, two anti-PD-1 antibodies—Nivolumab and Pembrolizumab—quickly received FDA approval for the treatment of metastatic melanoma, with comparable efficacy and toxicity profiles [84,85,86].

Subsequent investigations regarding the combination of immunotherapies (e.g., Nivolumab plus Ipilimumab [87]) and the combination of targeted therapies (e.g., Dabrafenib plus Trametinib [80]) demonstrated a significant improvement in the long-term prognosis of melanoma patients. A recent publication released the 10-year clinical outcomes from the CheckMate 067 trial, confirming Nivolumab’s survival advantage in advanced melanoma [88]. The researchers reported remarkable median melanoma-specific survival in the Nivolumab–Ipilimumab group (over 120 months with 37% patients still alive) compared with Nivolumab monotherapy (49.4 months) and Ipilimumab monotherapy (21.9 months) [88].

In 2020, investigators further explored the potential of triple combination therapy in BRAF-mutant melanomas. Gutzmer et al. concluded that the addition of anti-PD-L1 (Atezolizumab) to BRAF inhibitor (Vemurafenib) and MEK therapy (Cobimetinib) was safe with prolonged progression-free survival (PFS) of unresectable advanced melanoma. Median PFS was 15.1 months in the Atezolizumab–Vemurafenib–Cobimetinib group vs. 10.6 months in the Vemurafenib–Cobimetinib group [89]. Subsequent analyses showed that OS did not improve substantially with Atezolizumab–Vemurafenib–Cobimetinib [90]. Given the absence of the OS benefit, IMspire150 did not shift the guidelines for the treatment of metastatic melanoma. To better understand the optimal sequencing of combined immunotherapy and targeted therapy, researchers initiated different trials for targeted therapy and immunotherapy in BRAF-V600 mutant melanomas. In the SECOMBIT trial, investigators observed a higher overall response rate (ORR) when immunotherapy was given before BRAF/MEK inhibitors. A follow-up study further confirmed the improved survival benefit of immunotherapy as first-line vs. second-line therapy in *BRAF*-mutant metastatic melanoma (4-year OS of 64% vs. 46%). However, the SECOMBIT trial was not sufficiently powered to draw definitive conclusions due to small patient numbers [91]. Another similarly designed trial with a larger cohort of patients, DREAMseq, also supported the immunotherapy-first sequence. In the DREAMseq trial, immunotherapy showed superior efficacy over targeted therapy in treating naïve *BRAF*-mutant metastatic melanoma (5-year OS of 63% vs. 34%) [92]. These data highlight an unmet need to understand treatment resistance mechanisms, improve patient selection, and optimise treatment sequencing for better treatment decision-making.

Shortly after the approval of Nivolumab and Pembrolizumab, the first oncolytic viral immunotherapy, Talimogene laherparepvec (T-VEC), was approved for the treatment of unresectable melanoma. T-VEC is a genetically modified herpes simplex virus (HSV) type 1 that selectively lyses tumour cells and augments anti-tumour immune response [93]. Meta-analysis by Stahlie et al. concluded that single-agent T-VEC achieves impressive response rates with mild toxicities, especially in early metastatic (stage IIIB-IVM1a) melanoma (pooled ORR of 58%) [94]. Subsequent attempts in multimodal strategies showed mixed results, indicating the unexplored potential of T-VEC. For example, the combination with pembrolizumab (MASTERKEY-265) showed no additional benefits, whereas the combination with Nivolumab (NIVEC) or Ipilimumab was associated with added clinical benefits [95,96,97]. It is disappointing that MASTERKEY-265 has failed to meet its primary endpoint despite earlier success [98]. One possible explanation is that MASTERKEY-265 recruited a higher percentage of stage IIIB/C/IVM1a melanoma patients compared to the pivotal KEYNOTE-006 trial [99]. Another possibility is the less frequent T-VEC dosing in MASTERKEY-265 compared to OpTim (once every two weeks until week 9, then once every three weeks vs. once every two weeks) [93]. Therefore, the potential of T-VEC remains uncertain.

### 3.7. LAG-3 Inhibitor

In addition to CTLA-4 and PD-1, lymphocyte activation gene-3 (LAG-3) was found to be upregulated on activated T cells and negatively regulated their expansion [100]. Co-expression of LAG-3 and PD-1 in tumour-infiltrating lymphocytes was reported to mediate tumour-induced T cell exhaustion [101,102]. The first FDA-approved LAG-3 inhibitor (2022) is Relatlimab, which blocks the binding ability of LAG-3 to MHC-II ligands [103]. The first clinical study (RELATIVITY-047) employing Relatlimab and Nivolumab was released in early 2022. PFS in patients receiving the combined Relatlimab–Nivolumab drastically improved (10.1 months) in comparison to patients receiving Nivolumab as a single agent (4.6 months). A recent three-year follow-up report continued to show sustained benefits and a consistent safety profile, suggesting that dual inhibition of LAG-3 and PD-1 holds promise as a first-line treatment for metastatic melanoma [104,105].

### 3.8. Lifileucel

Following the recent therapeutic breakthroughs, a decline in melanoma mortality (1.4%) was observed from 2017 to 2022, compared with 6.1% per year from 2013 to 2017 [106]. In February 2024, the FDA approved an adoptive immune cell therapy using autologous ex vivo expanded tumour-infiltrating lymphocytes (TILs), known as Lifileucel (LN-144) (Amtagvi, Iovance Biotherapeutics, Inc., San Carlos, CA, USA), for unresectable and metastatic melanomas previously treated with immune checkpoint inhibitors and (if BRAF V600 was mutation-positive) BRAF/MEK inhibitors [107]. This accelerated approval was granted based on the findings from the C-144-01 trial (NCT02360579). In this study, the recruited patients were heavily pretreated with immune checkpoint inhibitors (anti PD-1/PD-L1/CTLA4). These patients had a limited response to chemotherapy, with only a 4–10% objective response rate (ORR) and a MOS of 7 months [108,109,110]. In the C-144-01 trial, patients receiving TIL achieved a remarkable ORR of 36%, supporting its potential as an alternative treatment in ICI-refractory melanoma [111].

## 4. Emerging Biomarkers

The efficacy of the aforementioned therapies largely depends on the status of genetic and immune-based biomarkers. The discovery of new biomarkers is thus pivotal to better stratify the patients and provide treatment alternatives for non-responders. Table 1 summarises several existing and rising biomarkers. Both Nicotinamide N-methyltransferase (NNMT) and Paraoxonase-2 (PON2) are enzymes that are overexpressed in melanoma lesions compared to benign nevi [112,113,114]. NNMT catalyses the N-methylation of nicotinamide and contributes to the detoxification of xenobiotic compounds [115]. NNMT was postulated to participate in early carcinogenesis and BRAF inhibitor (BRAFi) resistance, thus holding prognostic value [112,113,115]. Pharmacological inhibition and genetic silencing of NNMT have been shown to hamper BRAFi-resistant melanoma cell growth, demonstrating its potential as a therapeutic vulnerability [112]. On the other hand, PON2 protects cells against reactive oxygen species [114] and its silencing sensitises melanoma cells towards chemotherapy [116]. Another novel prognostic model is SKCM-P8, which stratifies patients (hypo- vs. hypermethylated) based on the methylation orderings of eight pairs of loci [117]. Although current preclinical evidence for SKCM-P8 is limited, it has been associated with immune infiltration, making it useful for predicting ICI response [117]. Lactate dehydrogenase (LDH) and S100B are independent metabolic prognostic markers in melanoma [21,118]. LDH catalyses the conversion of pyruvate to lactate, and is released upon cell death, indicating disease progression. Meanwhile, S100B inhibits p53-dependent cell apoptosis [119,120]. S100B, however, demonstrates greater sensitivity for early metastasis detection compared to LDH [121]. Finally, circulating tumour DNA (ctDNA), which is released by dying cancer cells, is also a powerful prognostic marker. For instance, in pretreatment BRAF V600 mutants, elevated ctDNA predicted worse OS independent of their LDH levels [122]. Since DNA methylation profile can change as melanoma progresses, the detection of ctDNA methylation could provide valuable insights into disease progression. Some methylated genes have been validated as biomarkers for melanoma, with respect to disease development (*HOXA9* methylation), progression (*TBC1D16* methylation), and prognosis *(PON3* and *OVOL1* methylation) [123].

## 5. Future Directions

Based on the timeline discussed above, there is a shift in the research focus aimed at prolonging the response duration of existing immune checkpoint inhibitors (ICIs) through combination strategies, as well as managing persistent or recurrent disease after ICI treatment.

Following the superior efficacy of dual ICI, we expect more interrogations into triplet ICI in the coming years. Preliminary results from trial RELATIVITY-048 evaluating triplet checkpoint blockade (Nivolumab–Relatlimab–Ipilimumab) in advanced melanoma and the case study in glioblastoma have shown convincing clinical benefits and warrant further research [129,130]. Furthermore, other promising immune checkpoint targets are currently under intense investigation, e.g., T cell immunoglobulin and mucin-domain-containing-3 (TIM-3), which is associated with T cell dysfunction. The AMBER Phase I trial has demonstrated the promising activity of the TIM-3 inhibitor, Cobolimab, in melanoma patients [131]. Cobolimab, in combination with the PD-1 inhibitor Dostarlimab, is currently in a Phase II clinical trial (NCT04139902) and has shown better outcomes for high-risk resectable melanomas [132]. Another emerging checkpoint target is TIGIT, a T cell immunoreceptor containing an immunoglobulin and Immunoreceptor tyrosine-based inhibitory motif (ITIM) domain, which activates the inhibitory machinery of T cells, NK cells, and regulatory T cells [133,134,135]. Combining the anti-TIGIT drug, Vibostolimab, with anti-PD-1 pembrolizumab has demonstrated improved outcomes in stage III melanoma patients compared to pembrolizumab alone, as reported by Dummer et al., in clinical trial NCT04303169 [136]. We also expect continued investigations into the combination of novel compounds/targeted therapy with ICI. A study by Pietrobono et al. showed that the Gentian Violet dye inhibits in vitro melanoma stem cell growth via SOX2, STAT3, and AKT downregulation while augmenting reactive oxygen species (ROS) [137]. They demonstrated that the previously reported antifungal/antibacterial compound and the mentioned signalling mechanisms could be further explored in melanoma therapy settings.

In addition, with the recent approval of Lifileucel, we are optimistic that Chimeric Antigen Receptor (CAR) therapies will continue to become available to melanoma patients through clinical trials. In CAR therapies, T cells or NK cells are engineered to express receptors against tumour-specific antigens before being expanded and infused into patients. Clinical trial of IL-13Rα2-targeted CAR-T cell therapy, for example, has shown convincing phase I results in glioblastoma (NCT02208362) [138]. Meanwhile, the testing of its efficacy in melanoma is currently underway (NCT04119024). In the pilot phase I trial examining cMET-targeted CAR-T therapy, two out of three stage III/IV melanomas achieved stable disease without grade 3/4 adverse events, thus warranting further investigation [139]. However, this trial (NCT03060356) was terminated due to funding constraints. On the other hand, a phase I study of GD2-targeted CAR-T in BRAF-mutant melanomas concluded that this regimen is safe but lacks persistence in CAR-T expansions (NCT02482532) [140]. The authors speculated that the lack of clinical benefits of GD2-targeted CAR-T may be attributed to short CAR-T cell persistence and impaired T cell functions by concurrent BRAF/MEK inhibitor treatment [140]. Despite its insufficiency to warrant a phase II study, this trial underscores that GD2-CAR-T approach remains promising. The group speculated that screening for biomarkers associated with CAR-T persistence and prior treatment profiling might better stratify patients into treatment responders and non-responders [140].

In contrast to CAR-T therapy, CAR-NK clinical trials are relatively fewer in number. This is likely due to its lower persistence in the body and less understanding compared to CAR-T therapy. Unlike CAR-T therapy, CAR-NK is advantageous because it does not induce graft-versus-host disease (GvHD) and possesses both CAR-dependent and -independent killing activities [141]. A preclinical study had shown that CAR-NK92-targeting CD276 exerts potent cytotoxic activity against 2D and 3D melanoma models [142]. Following the promising clinical outcomes of combined N-803 (IL-15 agonist) and PDL1-NK cell therapy in advanced pancreatic cancer patients (NCT04390399), research has been extended into other solid tumours [143], including melanoma (NCT04898543). Several other phase I CAR-NK studies include anti-5T4 (Oncofetal Trophoblast Glycoprotein)- CAR-NK (NCT05194709); NKG2D-CAR-NK92 (NCT05528341); and NKG2DL-CAR-NK (NCT03415100).

Nonetheless, the treatment efficacy of the mentioned immunotherapies is not uniform across all patients. This is especially the case in immunologically “cold” tumours, which lack tumour antigens and tumour-infiltrating lymphocytes. Such barriers have prompted researchers to explore emerging therapeutic technologies. Some of the up-and-coming treatment strategies include extracellular vesicle (EV)-based therapy, proteolysis-targeting chimaera (PROTAC), and nanoparticles.

EVs, such as exosomes, microvesicles, and apoptotic bodies, are naturally occurring transporters produced by various cell types and released into the extracellular environment [144]. Growing evidence suggests that exosomes contribute to immunosuppression through PD-L1 and IL-10 expression [145,146], promote melanoma invasion [147,148], and, to a lesser extent, promote tumour cell proliferation [149]. Specifically, Chen et al. observed an elevated circulating exosomal PD-L1 in anti-PD-1 non-responders [145]. This phenomenon was associated with worse clinical outcomes in melanoma patients. The group further demonstrated that melanoma cell-derived exosomes can bind to PD-1^+^ CD8 T cells, thereby inhibiting their cytotoxic functions [145]. Their findings corroborate our work, which shows that targeting exosome trafficking via RAB27A blockage impairs melanoma cell invasion in vitro [147]. Taken together, these investigations suggest that exosome-based strategies may enhance current immunotherapy, either by acting as a blood-based biomarker to indicate treatment responsiveness or by controlling tumour progression.

Alternatively, the ability of exosomes to transport molecular cargo, such as DNA, RNA, and microRNA, to other cells through phagocytosis could be exploited as a therapeutic delivery tool for cancer therapy [150]. Most studies aimed at exploiting exosome-based therapy for melanomas are limited to preclinical stages, as reviewed to date [151]. The only clinical trial data on dendritic cell-derived exosomes in melanoma were published in 2005, supporting the safety of exosome therapy [152]. Until additional data become available, the clinical benefits of exosome therapy remain ambiguous.

Another approach worth discussing is proteolysis-targeting chimaera (PROTAC)-based protein degrader treatment. PROTAC typically consists of two ligands connected by a linker: one binds to the target protein of interest, and the other one recruits E3 ubiquitin ligase. In this way, the target protein will be degraded by the ubiquitin–proteasome system (UPS) [153]. PROTAC is currently attracting substantial attention given its profound target degradation ability and minimal off-target effects. There is currently no FDA-approved PROTAC, but it is anticipated that Vepdegestrant, an estrogen receptor degrader, will be approved for the treatment of breast cancer by 2027 [154]. Two additional PROTACs currently in Phase III trials are CC-94676 (BMS-986365) and BGB-16673, targeting the androgen receptor and Bruton’s tyrosine kinase, respectively (NCT06764485, NCT06846671). Preclinical data in melanoma have demonstrated improved anti-tumour activity when PROTAC was employed compared to traditional inhibitors against BRAF [155,156,157,158]. These data highlight the potential of next-generation of targeted degraders against aberrantly expressed molecules in melanoma, such as MEK, ERK, and CDK4/6. As mentioned earlier, the *CDKN2A* mutation leading to p16 loss is a frequent event in melanoma. Numerous preclinical studies have shown that CDK4/6 inhibitors, e.g., palbociclib, ribociclib, and abemaciclib, can restore the functional consequences of p16 loss and impede melanoma growth [159]. Interestingly, CDK4/6 inhibition also sensitises melanoma patients towards immunotherapy [160]. This response may be partly due to enhanced antigen processing and presentation within the tumour microenvironment, as demonstrated in breast cancer in vitro and in vivo [161].

Employing nanotechnology is another promising strategy for improving the efficacy of targeted cancer therapies, which includes liposomes, dendrimers, carbon nanotubes, and metal nanoparticles. The advantages of nanotechnology include mitigating drug resistance and facilitating drug uptake [162]. Unfortunately, the complex nanoparticle formulations render the mass production expensive, and unexpected immune complications post-administration remain a concern [163]. To date, there is no FDA-approved nanotherapy for melanoma.

## 6. Conclusions

In conclusion, immune checkpoint inhibitors (ICIs) remain the most effective treatment option for the management of cutaneous melanoma. Targeted therapies such as BRAF and MEK inhibitors are effective first-line treatments for patients with specific oncogenic mutations. Based on Phase III clinical trials and meta-analyses, anti-PD-1/PD-L1 therapies represent the most effective ICIs for melanoma. Anti-CTLA-4 is also effective but often presents with severe immune-related side effects. Anti-LAG3 shows great potential in combination with anti-PD-1/PD-L1 as a first-line treatment. Numerous studies are exploring combining different ICIs or ICIs with targeted therapies to improve the overall outcome of melanoma patients.

Although CAR-T or CAR-NK therapy showed some benefits in early-phase clinical trials, their potential as melanoma treatments is still too early to predict. PROTAC-based therapies, e.g., BRAF-targeting degraders, have shown promising early results but require further investigation. Extracellular vesicle (EV)-based treatments also warrant further investigation, although they remain largely at the preclinical stage.

Overall, ICI has established a high benchmark for melanoma treatment, with tremendous future potential in multimodal therapies. Advancements in biomarker identification, drug resistance mechanisms, and interdisciplinary collaborations will continue to refine the treatment plans for patients in the coming years.

## Figures and Tables

**Table 1:** Existing and emerging biomarkers in melanoma.

Category	Biomarker Name	Key Association	Mechanism	References
Genetic and Molecular	BRAF, NRAS, MEK NF1, KIT, PTEN, TERT	Predict response to targeted therapy	Regulate MAPK pathway; act as a tumour suppressor; regulate telomerase	[10,12,124,125,126]
Immune	PD-1, PD-L1, CTLA-4, LAG3, TIGIT, TILs	Predict response to ICIs	Modulate immune evasion	[61,83,84,101,102]
Epigenetic	SKCM-P8, NNMT	Predict disease aggressiveness; predict chemosensitivity; regulates immune checkpoints	Regulate chemosensitivity	[112,113,115,117]
Metabolic	PON2, S100B, LDH	Predict disease aggressiveness and recurrence; predict chemosensitivity; necrosis and metastasis marker	Protects against reactive oxygen species; regulates pyruvate to lactate conversion; inhibits p53-dependent apoptosis	[114,116,118,120,121,127]
Liquid Biopsy	ctDNA, methylated ctDNA, miRNA	Predict response to targeted therapy	Modulate cell/tissue polarity, adhesion, and inflammatory processes	[122,123,128]

## Data Availability

Data are contained within the article.

## References

[1] Domingues B., Lopes J.M., Soares P., Populo H. (2018). Melanoma treatment in review. Immunotargets Ther..

[2] Matthews N.H., Li W.Q., Qureshi A.A., Weinstock M.A., Cho E., Ward W.H., Farma J.M. (2017). Epidemiology of Melanoma. Cutaneous Melanoma: Etiology and Therapy.

[3] Long G.V., Swetter S.M., Menzies A.M., Gershenwald J.E., Scolyer R.A. (2023). Cutaneous melanoma. Lancet.

[4] Tasdogan A., Sullivan R.J., Katalinic A., Lebbe C., Whitaker D., Puig S., van de Poll-Franse L.V., Massi D., Schadendorf D. (2025). Cutaneous melanoma. Nat. Rev. Dis. Primers.

[5] Newell F., Johansson P.A., Wilmott J.S., Nones K., Lakis V., Pritchard A.L., Lo S.N., Rawson R.V., Kazakoff S.H., Colebatch A.J. (2022). Comparative Genomics Provides Etiologic and Biological Insight into Melanoma Subtypes. Cancer Discov..

[6] Kaliki S., Shields C.L. (2017). Uveal melanoma: Relatively rare but deadly cancer. Eye.

[7] Gonzalez-Cao M., Berciano-Guerrero M.-Á., Muñoz-Couselo E., Manzano J.L., Cerezuela-Fuentes P., Crespo G., Soria A., de Miguel P.A., Gutiérrez Sanz L., de la Rosa C.A. (2026). Poor efficacy of anti PD-1 antibody based immunotherapy in patients with acral melanoma: Results from the Spanish Melanoma Group (GEM) registry. Clin. Transl. Oncol..

[8] Tod B., Esterhuizen T., Visser W., Kotze M., Bowcock A., Schneider J., Burger H. (2025). Survival Outcomes of a Large Cohort of Acral Melanoma Patients Treated at a South African Referral Hospital. J. Ski. Cancer.

[9] Stålhammar G., Herrspiegel C. (2022). Long-term relative survival in uveal melanoma: A systematic review and meta-analysis. Commun. Med..

[10] Akbani R., Akdemir K.C., Aksoy B.A., Albert M., Ally A., Amin S.B., Arachchi H., Arora A., Auman J.T., Ayala B. (2015). Genomic classification of cutaneous melanoma. Cell.

[11] Klinac D., Gray E.S., Millward M., Ziman M. (2013). Advances in personalized targeted treatment of metastatic melanoma and non-invasive tumor monitoring. Front. Oncol..

[12] Curtin J.A., Fridlyand J., Kageshita T., Patel H.N., Busam K.J., Kutzner H., Cho K.-H., Aiba S., Bröcker E.-B., LeBoit P.E. (2005). Distinct sets of genetic alterations in melanoma. N. Engl. J. Med..

[13] Davies H., Bignell G.R., Cox C., Stephens P., Edkins S., Clegg S., Teague J., Woffendin H., Garnett M.J., Bottomley W. (2002). Mutations of the BRAF gene in human cancer. Nature.

[14] Rubinstein J.C., Sznol M., Pavlick A.C., Ariyan S., Cheng E., Bacchiocchi A., Kluger H.M., Narayan D., Halaban R. (2010). Incidence of the V600K mutation among melanoma patients with BRAF mutations, and potential therapeutic response to the specific BRAF inhibitor PLX4032. J. Transl. Med..

[15] Flaherty K.T., Puzanov I., Kim K.B., Ribas A., McArthur G.A., Sosman J.A., O’Dwyer P.J., Lee R.J., Grippo J.F., Nolop K. (2010). Inhibition of mutated, activated BRAF in metastatic melanoma. N. Engl. J. Med..

[16] McArthur G.A., Chapman P.B., Robert C., Larkin J., Haanen J.B., Dummer R., Ribas A., Hogg D., Hamid O., Ascierto P.A. (2014). Safety and efficacy of vemurafenib in BRAF V600E and BRAF V600K mutation-positive melanoma (BRIM-3): Extended follow-up of a phase 3, randomised, open-label study. Cancer Discov..

[17] Flaherty K.T., Hennig M., Lee S.J., Ascierto P.A., Dummer R., Eggermont A.M., Hauschild A., Kefford R., Kirkwood J.M., Long G.V. (2014). Surrogate endpoints for overall survival in metastatic melanoma: A meta-analysis of randomised controlled trials. Lancet Oncol..

[18] Long G.V., Stroyakovskiy D., Gogas H., Levchenko E., De Braud F., Larkin J., Garbe C., Jouary T., Hauschild A., Grob J.J. (2014). Combined BRAF and MEK inhibition versus BRAF inhibition alone in melanoma. N. Engl. J. Med..

[19] Robert C., Karaszewska B., Schachter J., Rutkowski P., Mackiewicz A., Stroiakovski D., Lichinitser M., Dummer R., Grange F., Mortier L. (2015). Improved overall survival in melanoma with combined dabrafenib and trametinib. N. Engl. J. Med..

[20] Switzer B., Puzanov I., Skitzki J.J., Hamad L., Ernstoff M.S. (2022). Managing Metastatic Melanoma in 2022: A Clinical Review. JCO Oncol. Pract..

[21] Balch C.M., Gershenwald J.E., Soong S.J., Thompson J.F., Atkins M.B., Byrd D.R., Buzaid A.C., Cochran A.J., Coit D.G., Ding S. (2009). Final version of 2009 AJCC melanoma staging and classification. J. Clin. Oncol..

[22] Gershenwald J.E., Scolyer R.A. (2018). Melanoma Staging: American Joint Committee on Cancer (AJCC) 8th Edition and Beyond. Ann. Surg. Oncol..

[23] Hamid O., Robert C., Daud A., Hodi F.S., Hwu W.J., Kefford R., Wolchok J.D., Hersey P., Joseph R.W., Weber J.S. (2013). Safety and tumor responses with lambrolizumab (anti-PD-1) in melanoma. N. Engl. J. Med..

[24] Hodi F.S., O’Day S.J., McDermott D.F., Weber R.W., Sosman J.A., Haanen J.B., Gonzalez R., Robert C., Schadendorf D., Hassel J.C. (2010). Improved survival with ipilimumab in patients with metastatic melanoma. N. Engl. J. Med..

[25] Larkin J., Chiarion-Sileni V., Gonzalez R., Grob J.J., Rutkowski P., Lao C.D., Cowey C.L., Schadendorf D., Wagstaff J., Dummer R. (2019). Five-Year Survival with Combined Nivolumab and Ipilimumab in Advanced Melanoma. N. Engl. J. Med..

[26] Long G.V., Hauschild A., Santinami M., Atkinson V., Mandalà M., Chiarion-Sileni V., Larkin J., Nyakas M., Dutriaux C., Haydon A. (2017). Adjuvant Dabrafenib plus Trametinib in Stage III BRAF-Mutated Melanoma. N. Engl. J. Med..

[27] Hauschild A., Dummer R., Santinami M., Atkinson V., Mandala M., Merelli B., Chiarion-Sileni V., Haydon A.M., Schachter J., Schadendorf D. (2024). Long-term follow up for adjuvant dabrafenib plus trametinib in stage III BRAF-mutated melanoma: Final results of the COMBI-AD study. J. Clin. Oncol..

[28] Tikoo S., Jain R., Tomasetig F., On K., Martinez B., Heu C., Stehle D., Obeidy P., Guo D., Vincent J.N. (2021). Amelanotic B16-F10 Melanoma Compatible with Advanced Three-Dimensional Imaging Modalities. J. Investig. Dermatol..

[29] Guo D., Jurek R., Beaumont K.A., Sharp D.S., Tan S.Y., Mariana A., Failes T.W., Grootveld A.K., Bhattacharyya N.D., Phan T.G. (2023). Invasion-Block and S-MARVEL: A high-content screening and image analysis platform identifies ATM kinase as a modulator of melanoma invasion and metastasis. Proc. Natl. Acad. Sci. USA.

[30] Carter S.K., Friedman M.A. (1972). 5-(3,3-dimethyl-1-triazeno)-imidazole-4-carboxamide (DTIC, DIC, NSC-45388)—A new antitumor agent with activity against malignant melanoma. Eur. J. Cancer.

[31] Shealy Y.F., Montgomery J.A., Laster W.R. (1962). Antitumor activity of triazenoimidazoles. Biochem. Pharmacol..

[32] Kim C., Lee C.W., Kovacic L., Shah A., Klasa R., Savage K.J. (2010). Long-term survival in patients with metastatic melanoma treated with DTIC or temozolomide. Oncologist.

[33] Hill G.J., Krementz E.T., Hill H.Z. (1984). Dimethyl triazeno imidazole carboxamide and combination therapy for melanoma. IV. Late results after complete response to chemotherapy (Central Oncology Group protocols 7130, 7131, and 7131A). Cancer.

[34] Lui P., Cashin R., Machado M., Hemels M., Corey-Lisle P.K., Einarson T.R. (2007). Treatments for metastatic melanoma: Synthesis of evidence from randomized trials. Cancer Treat. Rev..

[35] Kirkwood J.M., Strawderman M.H., Ernstoff M.S., Smith T.J., Borden E.C., Blum R.H. (1996). Interferon alfa-2b adjuvant therapy of high-risk resected cutaneous melanoma: The Eastern Cooperative Oncology Group Trial EST 1684. J. Clin. Oncol..

[36] Grossmann K.F., Othus M., Patel S.P., Tarhini A.A., Sondak V.K., Knopp M., Petrella T.M., Truong T.G., Khushalani N., Cohen J. (2022). Adjuvant Pembrolizumab versus IFNα2b or Ipilimumab in Resected High-Risk Melanoma. CANCER Discov..

[37] Larkin J., Del Vecchio M., Mandalá M., Gogas H., Arance Fernandez A.M., Dalle S., Cowey C.L., Schenker M., Grob J.J., Chiarion-Sileni V. (2023). Adjuvant Nivolumab versus Ipilimumab in Resected Stage III/IV Melanoma: 5-Year Efficacy and Biomarker Results from CheckMate 238. Clin. Cancer Res..

[38] Raeber M.E., Sahin D., Karakus U., Boyman O. (2023). A systematic review of interleukin-2-based immunotherapies in clinical trials for cancer and autoimmune diseases. eBioMedicine.

[39] Hesketh P., Caguioa P., Koh H., Dewey H., Facada A., McCaffrey R., Parker K., Nylen P., Woodworth T. (1993). Clinical activity of a cytotoxic fusion protein in the treatment of cutaneous T-cell lymphoma. J. Clin. Oncol..

[40] Foss F.M., Borkowski T.A., Gilliom M., Stetler-Stevenson M., Jaffe E.S., Figg W.D., Tompkins A., Bastian A., Nylen P., Woodworth T. (1994). Chimeric fusion protein toxin DAB486IL-2 in advanced mycosis fungoides and the Sezary syndrome: Correlation of activity and interleukin-2 receptor expression in a phase II study. Blood.

[41] Liu C., Workman C.J., Vignali D.A. (2016). Targeting regulatory T cells in tumors. FEBS J..

[42] Whiteside S.K., Grant F.M., Alvisi G., Clarke J., Tang L., Imianowski C.J., Zhang B., Evans A.C., Wesolowski A.J., Conti A.G. (2023). Acquisition of suppressive function by conventional T cells limits antitumor immunity upon T(reg) depletion. Sci. Immunol..

[43] Jones E., Dahm-Vicker M., Simon A.K., Green A., Powrie F., Cerundolo V., Gallimore A. (2002). Depletion of CD25+ regulatory cells results in suppression of melanoma growth and induction of autoreactivity in mice. Cancer Immun..

[44] Golgher D., Jones E., Powrie F., Elliott T., Gallimore A. (2002). Depletion of CD25+ regulatory cells uncovers immune responses to shared murine tumor rejection antigens. Eur. J. Immunol..

[45] Attia P., Maker A.V., Haworth L.R., Rogers-Freezer L., Rosenberg S.A. (2005). Inability of a Fusion Protein of IL-2 and Diphtheria Toxin (Denileukin Diftitox, DAB389IL-2, ONTAK) to Eliminate Regulatory T Lymphocytes in Patients with Melanoma. J. Immunother..

[46] Rasku M.A., Clem A.L., Telang S., Taft B., Gettings K., Gragg H., Cramer D., Lear S.C., McMasters K.M., Miller D.M. (2008). Transient T cell depletion causes regression of melanoma metastases. J. Transl. Med..

[47] Telang S., Rasku M.A., Clem A.L., Carter K., Klarer A.C., Badger W.R., Milam R.A., Rai S.N., Pan J., Gragg H. (2011). Phase II trial of the regulatory T cell-depleting agent, denileukin diftitox, in patients with unresectable stage IV melanoma. BMC Cancer.

[48] Kawai H., Ando K., Maruyama D., Yamamoto K., Kiyohara E., Terui Y., Fukuhara N., Miyagaki T., Tokura Y., Sakata-Yanagimoto M. (2021). Phase II study of E7777 in Japanese patients with relapsed/refractory peripheral and cutaneous T-cell lymphoma. Cancer Sci..

[49] Tsai J., Lee J.T., Wang W., Zhang J., Cho H., Mamo S., Bremer R., Gillette S., Kong J., Haass N.K. (2008). Discovery of a selective inhibitor of oncogenic B-Raf kinase with potent antimelanoma activity. Proc. Natl. Acad. Sci. USA.

[50] Lee J.T., Li L., Brafford P.A., van den Eijnden M., Halloran M.B., Sproesser K., Haass N.K., Smalley K.S.M., Tsai J., Bollag G. (2010). PLX4032, a potent inhibitor of the B-Raf V600E oncogene, selectively inhibits V600E-positive melanomas. Pigment Cell Melanoma Res..

[51] Ribas A., Kim K.B., Schuchter L.M., Gonzalez R., Pavlick A.C., Weber J.S., McArthur G.A., Hutson T.E., Flaherty K.T., Moschos S.J. (2011). BRIM-2: An open-label, multicenter phase II study of vemurafenib in previously treated patients with *BRAF* V600E mutation-positive metastatic melanoma. J. Clin. Oncol..

[52] Chapman P.B., Hauschild A., Robert C., Haanen J.B., Ascierto P., Larkin J., Dummer R., Garbe C., Testori A., Maio M. (2011). Improved Survival with Vemurafenib in Melanoma with BRAF V600E Mutation. N. Engl. J. Med..

[53] Schilling B., Sondermann W., Zhao F., Griewank K.G., Livingstone E., Sucker A., Zelba H., Weide B., Trefzer U., Wilhelm T. (2014). Differential influence of vemurafenib and dabrafenib on patients’ lymphocytes despite similar clinical efficacy in melanoma. Ann. Oncol..

[54] Jhaveri K.D., Sakhiya V., Fishbane S. (2015). Nephrotoxicity of the BRAF Inhibitors Vemurafenib and Dabrafenib. JAMA Oncol..

[55] Bai Y., Kim J.Y., Bisunke B., Jayne L.A., Silvaroli J.A., Balzer M.S., Gandhi M., Huang K.M., Sander V., Prosek J. (2021). Kidney toxicity of the BRAF-kinase inhibitor vemurafenib is driven by off-target ferrochelatase inhibition. Kidney Int..

[56] Menzies A.M., Long G.V. (2014). Dabrafenib and trametinib, alone and in combination for BRAF-mutant metastatic melanoma. Clin. Cancer Res..

[57] Ballantyne A.D., Garnock-Jones K.P. (2013). Dabrafenib: First Global Approval. Drugs.

[58] Radi R., Huang C., Elsey J., Jung Y.H., Corces V.G., Arbiser J.L. (2022). Indolium 1 Exerts Activity against Vemurafenib-Resistant Melanoma In Vivo. Antioxidants.

[59] Ballas Z.K. (2018). The 2018 Nobel Prize in Physiology or Medicine: An exemplar of bench to bedside in immunology. J. Allergy Clin. Immunol..

[60] Krummel M.F., Allison J.P. (1995). CD28 and CTLA-4 have opposing effects on the response of T cells to stimulation. J. Exp. Med..

[61] Leach D.R., Krummel M.F., Allison J.P. (1996). Enhancement of Antitumor Immunity by CTLA-4 Blockade. Science.

[62] Wolchok J.D., Neyns B., Linette G., Negrier S., Lutzky J., Thomas L., Waterfield W., Schadendorf D., Smylie M., Guthrie T. (2010). Ipilimumab monotherapy in patients with pretreated advanced melanoma: A randomised, double-blind, multicentre, phase 2, dose-ranging study. Lancet Oncol..

[63] Hersh E.M., O’Day S.J., Powderly J., Khan K.D., Pavlick A.C., Cranmer L.D., Samlowski W.E., Nichol G.M., Yellin M.J., Weber J.S. (2011). A phase II multicenter study of ipilimumab with or without dacarbazine in chemotherapy-naïve patients with advanced melanoma. Investig. New Drugs.

[64] Robert C., Thomas L., Bondarenko I., O’Day S., Weber J., Garbe C., Lebbe C., Baurain J.-F., Testori A., Grob J.-J. (2011). Ipilimumab plus Dacarbazine for Previously Untreated Metastatic Melanoma. N. Engl. J. Med..

[65] Bertrand A., Kostine M., Barnetche T., Truchetet M.E., Schaeverbeke T. (2015). Immune related adverse events associated with anti-CTLA-4 antibodies: Systematic review and meta-analysis. BMC Med..

[66] Martins F., Sofiya L., Sykiotis G.P., Lamine F., Maillard M., Fraga M., Shabafrouz K., Ribi C., Cairoli A., Guex-Crosier Y. (2019). Adverse effects of immune-checkpoint inhibitors: Epidemiology, management and surveillance. Nat. Rev. Clin. Oncol..

[67] Smalley K.S., Haass N.K., Brafford P.A., Lioni M., Flaherty K.T., Herlyn M. (2006). Multiple signaling pathways must be targeted to overcome drug resistance in cell lines derived from melanoma metastases. Mol. Cancer Ther..

[68] Haass N.K., Sproesser K., Nguyen T.K., Contractor R., Medina C.A., Nathanson K.L., Herlyn M., Smalley K.S.M. (2008). The Mitogen-Activated Protein/Extracellular Signal-Regulated Kinase Kinase Inhibitor AZD6244 (ARRY-142886) Induces Growth Arrest in Melanoma Cells and Tumor Regression When Combined with Docetaxel. Clin. Cancer Res..

[69] Flaherty K.T., Robert C., Hersey P., Nathan P., Garbe C., Milhem M., Demidov L.V., Hassel J.C., Rutkowski P., Mohr P. (2012). Improved Survival with MEK Inhibition in BRAF-Mutated Melanoma. N. Engl. J. Med..

[70] Wang X., Luo Z., Chen J., Chen Y., Ji D., Fan L., Chen L., Zhao Q., Hu P., Sun P. (2023). First-in-human phase I dose-escalation and dose-expansion trial of the selective MEK inhibitor HL-085 in patients with advanced melanoma harboring NRAS mutations. BMC Med..

[71] Schreuer M., Jansen Y., Planken S., Chevolet I., Seremet T., Kruse V., Neyns B. (2017). Combination of dabrafenib plus trametinib for BRAF and MEK inhibitor pretreated patients with advanced BRAFV600-mutant melanoma: An open-label, single arm, dual-centre, phase 2 clinical trial. Lancet Oncol..

[72] Yamaguchi T., Yoshida T., Kurachi R., Kakegawa J., Hori Y., Nanayama T., Hayakawa K., Abe H., Takagi K., Matsuzaki Y. (2007). Identification of JTP-70902, a p15INK4b-inductive compound, as a novel MEK1/2 inhibitor. Cancer Sci..

[73] Mansour S.J., Matten W.T., Hermann A.S., Candia J.M., Rong S., Fukasawa K., Vande Woude G.F., Ahn N.G. (1994). Transformation of mammalian cells by constitutively active MAP kinase kinase. Science.

[74] Hoshino R., Chatani Y., Yamori T., Tsuruo T., Oka H., Yoshida O., Shimada Y., Ari-i S., Wada H., Fujimoto J. (1999). Constitutive activation of the 41-/43-kDa mitogen-activated protein kinase signaling pathway in human tumors. Oncogene.

[75] Sebolt-Leopold J.S., Herrera R. (2004). Targeting the mitogen-activated protein kinase cascade to treat cancer. Nat. Rev. Cancer.

[76] Falchook G.S., Lewis K.D., Infante J.R., Gordon M.S., Vogelzang N.J., DeMarini D.J., Sun P., Moy C., Szabo S.A., Roadcap L.T. (2012). Activity of the oral MEK inhibitor trametinib in patients with advanced melanoma: A phase 1 dose-escalation trial. Lancet Oncol..

[77] Subbiah V., Baik C., Kirkwood J.M. (2020). Clinical Development of BRAF plus MEK Inhibitor Combinations. Trends Cancer.

[78] Flaherty K.T., Infante J.R., Daud A., Gonzalez R., Kefford R.F., Sosman J., Hamid O., Schuchter L., Cebon J., Ibrahim N. (2012). Combined BRAF and MEK Inhibition in Melanoma with BRAF V600 Mutations. N. Engl. J. Med..

[79] Seth R., Agarwala S.S., Messersmith H., Alluri K.C., Ascierto P.A., Atkins M.B., Bollin K., Chacon M., Davis N., Faries M.B. (2023). Systemic Therapy for Melanoma: ASCO Guideline Update. J. Clin. Oncol..

[80] Robert C., Grob J.J., Stroyakovskiy D., Karaszewska B., Hauschild A., Levchenko E., Chiarion Sileni V., Schachter J., Garbe C., Bondarenko I. (2019). Five-Year Outcomes with Dabrafenib plus Trametinib in Metastatic Melanoma. N. Engl. J. Med..

[81] Nishimura H., Nose M., Hiai H., Minato N., Honjo T. (1999). Development of Lupus-like Autoimmune Diseases by Disruption of the PD-1 Gene Encoding an ITIM Motif-Carrying Immunoreceptor. Immunity.

[82] Ishida Y., Agata Y., Shibahara K., Honjo T. (1992). Induced expression of PD-1, a novel member of the immunoglobulin gene superfamily, upon programmed cell death. EMBO J..

[83] Iwai Y., Ishida M., Tanaka Y., Okazaki T., Honjo T., Minato N. (2002). Involvement of PD-L1 on tumor cells in the escape from host immune system and tumor immunotherapy by PD-L1 blockade. Proc. Natl. Acad. Sci. USA.

[84] Wang C., Thudium K.B., Han M., Wang X.-T., Huang H., Feingersh D., Garcia C., Wu Y., Kuhne M., Srinivasan M. (2014). In Vitro Characterization of the Anti-PD-1 Antibody Nivolumab, BMS-936558, and In Vivo Toxicology in Non-Human Primates. Cancer Immunol. Res..

[85] Robert C., Ribas A., Wolchok J.D., Hodi F.S., Hamid O., Kefford R., Weber J.S., Joshua A.M., Hwu W.J., Gangadhar T.C. (2014). Anti-programmed-death-receptor-1 treatment with pembrolizumab in ipilimumab-refractory advanced melanoma: A randomised dose-comparison cohort of a phase 1 trial. Lancet.

[86] Cybulska-Stopa B., Piejko K., Ostaszewski K., Dziura R., Galus Ł., Ziółkowska B., Kempa-Kamińska N., Ziętek M., Bal W., Kamycka A. (2023). Long-term clinical evidence of comparable efficacy and toxicity of nivolumab and pembrolizumab in advanced melanoma treatment. Melanoma Res..

[87] Alegre-del-Rey E.J., de la Nogal Fernandez B., Briceno-Casado P. (2017). Nivolumab and Ipilimumab in Advanced Melanoma. N. Engl. J. Med..

[88] Wolchok J.D., Chiarion-Sileni V., Rutkowski P., Cowey C.L., Schadendorf D., Wagstaff J., Queirolo P., Dummer R., Butler M.O., Hill A.G. (2025). Final, 10-Year Outcomes with Nivolumab plus Ipilimumab in Advanced Melanoma. N. Engl. J. Med..

[89] Gutzmer R., Stroyakovskiy D., Gogas H., Robert C., Lewis K., Protsenko S., Pereira R.P., Eigentler T., Rutkowski P., Demidov L. (2020). Atezolizumab, vemurafenib, and cobimetinib as first-line treatment for unresectable advanced BRAF(V600) mutation-positive melanoma (IMspire150): Primary analysis of the randomised, double-blind, placebo-controlled, phase 3 trial. Lancet.

[90] Ascierto P.A., Stroyakovskiy D., Gogas H., Robert C., Lewis K., Protsenko S., Pereira R.P., Eigentler T., Rutkowski P., Demidov L. (2023). Overall survival with first-line atezolizumab in combination with vemurafenib and cobimetinib in BRAF(V600) mutation-positive advanced melanoma (IMspire150): Second interim analysis of a multicentre, randomised, phase 3 study. Lancet Oncol..

[91] Chandra S., Choi J.S., Sosman J.A. (2023). Melanoma: Does Sequencing Really Matter?. J. Clin. Oncol..

[92] Atkins M.B., Lee S.J., Chmielowski B., Tarhini A.A., Cohen G.I., Gibney G.T., Truong T.-G., Davar D., Stephenson J., Curti B.D. (2025). DREAMseq: A phase III trial of treatment sequences in BRAFV600-mutant (m) metastatic melanoma (MM)—Final clinical results. J. Clin. Oncol..

[93] Andtbacka R.H.I., Kaufman H.L., Collichio F., Amatruda T., Senzer N., Chesney J., Delman K.A., Spitler L.E., Puzanov I., Agarwala S.S. (2015). Talimogene Laherparepvec Improves Durable Response Rate in Patients with Advanced Melanoma. J. Clin. Oncol..

[94] Stahlie E.H.A., Mulder E.E.A.P., Reijers S., Balduzzi S., Zuur C.L., Klop W.M.C., van der Hiel B., Van de Wiel B.A., Wouters M.W.J.M., Schrage Y.M. (2022). Single agent Talimogene Laherparepvec for stage IIIB-IVM1c melanoma patients: A systematic review and meta-analysis. Crit. Rev. Oncol./Hematol..

[95] Ribas A., Chesney J., Long G.V., Kirkwood J.M., Dummer R., Puzanov I., Hoeller C., Gajewski T.F., Gutzmer R., Rutkowski P. (2021). 1037O MASTERKEY-265: A phase III, randomized, placebo (Pbo)-controlled study of talimogene laherparepvec (T) plus pembrolizumab (P) for unresectable stage IIIB–IVM1c melanoma (MEL). Ann. Oncol..

[96] Zijlker L.P., Houdt W.J.v., Stahlie E.H.A., Franke V., Rohaan M.W., Delatzakis A., Zuur C., Klop W.M.C., Wiel B.A.v.d., Kuijpers A. (2023). Neoadjuvant T-VEC + nivolumab combination therapy for resectable early metastatic (stage IIIB/C/D-IV M1a) melanoma with injectable disease: NIVEC trial. J. Clin. Oncol..

[97] Chesney J.A., Puzanov I., Collichio F.A., Singh P., Milhem M.M., Glaspy J., Hamid O., Ross M., Friedlander P., Garbe C. (2023). Talimogene laherparepvec in combination with ipilimumab versus ipilimumab alone for advanced melanoma: 5-year final analysis of a multicenter, randomized, open-label, phase II trial. J. Immunother. Cancer.

[98] Long G., Dummer R., Johnson D., Michielin O., Martin-Algarra S., Treichel S., Chan E., Diede S., Ribas A. (2020). 429 Long-term analysis of MASTERKEY-265 phase 1b trial of talimogene laherparepvec (T-VEC) plus pembrolizumab in patients with unresectable stage IIIB-IVM1c melanoma. J. Immunother. Cancer.

[99] Robert C., Schachter J., Long G.V., Arance A., Grob J.J., Mortier L., Daud A., Carlino M.S., McNeil C., Lotem M. (2015). Pembrolizumab versus Ipilimumab in Advanced Melanoma. N. Engl. J. Med..

[100] Workman C.J., Vignali D.A. (2003). The CD4-related molecule, LAG-3 (CD223), regulates the expansion of activated T cells. Eur. J. Immunol..

[101] Woo S.R., Turnis M.E., Goldberg M.V., Bankoti J., Selby M., Nirschl C.J., Bettini M.L., Gravano D.M., Vogel P., Liu C.L. (2012). Immune inhibitory molecules LAG-3 and PD-1 synergistically regulate T-cell function to promote tumoral immune escape. Cancer Res..

[102] Anderson A.C., Joller N., Kuchroo V.K. (2016). Lag-3, Tim-3, and TIGIT: Co-inhibitory Receptors with Specialized Functions in Immune Regulation. Immunity.

[103] Su J., Fu Y., Cui Z., Abidin Z., Yuan J., Zhang X., Li R., Zhao C. (2023). Relatlimab: A novel drug targeting immune checkpoint LAG-3 in melanoma therapy. Front. Pharmacol..

[104] Tawbi H.A., Schadendorf D., Lipson E.J., Ascierto P.A., Matamala L., Castillo Gutierrez E., Rutkowski P., Gogas H.J., Lao C.D., De Menezes J.J. (2022). Relatlimab and Nivolumab versus Nivolumab in Untreated Advanced Melanoma. N. Engl. J. Med..

[105] Tawbi H.A., Hodi F.S., Lipson E.J., Schadendorf D., Ascierto P.A., Matamala L., Gutiérrez E.C., Rutkowski P., Gogas H., Lao C.D. (2024). Three-Year Overall Survival with Nivolumab Plus Relatlimab in Advanced Melanoma from RELATIVITY-047. J. Clin. Oncol..

[106] Siegel R.L., Kratzer T.B., Giaquinto A.N., Sung H., Jemal A. (2025). Cancer statistics, 2025. CA A Cancer J. Clin..

[107] Parums D.V. (2024). Editorial: First Regulatory Approval for Adoptive Cell Therapy with Autologous Tumor-Infiltrating Lymphocytes (TILs)—Lifileucel (Amtagvi). Med. Sci. Monit..

[108] Goldinger S.M., Lo S., Hassel J.C., Forschner A., McKean M.A., Zimmer L., Khoo C.C.H., Dummer R., Eroglu Z., Buchbinder E.I. (2018). The utility of chemotherapy after immunotherapy failure in metastatic melanoma: A multicenter case series. J. Clin. Oncol..

[109] Weichenthal M., Ugurel S., Leiter U.M., Satzger I., Kähler K.C., Welzel J., Pföhler C., Feldmann-Böddeker I., Meier F.E., Terheyden P. (2019). Salvage therapy after failure from anti-PD-1 single agent treatment: A Study by the German ADOReg melanoma registry. J. Clin. Oncol..

[110] Ribas A., Puzanov I., Dummer R., Schadendorf D., Hamid O., Robert C., Hodi F.S., Schachter J., Pavlick A.C., Lewis K.D. (2015). Pembrolizumab versus investigator-choice chemotherapy for ipilimumab-refractory melanoma (KEYNOTE-002): A randomised, controlled, phase 2 trial. Lancet Oncol..

[111] Sarnaik A.A., Hamid O., Khushalani N.I., Lewis K.D., Medina T., Kluger H.M., Thomas S.S., Domingo-Musibay E., Pavlick A.C., Whitman E.D. (2021). Lifileucel, a Tumor-Infiltrating Lymphocyte Therapy, in Metastatic Melanoma. J. Clin. Oncol..

[112] Ghezzi B., Fiorilla I., Carreira Á., Recco F., Sorci L., Avalle L., Ponzano A., Mazzola F., Todesco A.M., Tommasi N. (2025). NAMPT and NNMT released via extracellular vesicles and as soluble mediators are distinguished traits of BRAF inhibitor resistance of melanoma cells impacting on the tumor microenvironment. Cell Commun. Signal..

[113] Ganzetti G., Sartini D., Campanati A., Rubini C., Molinelli E., Brisigotti V., Cecati M., Pozzi V., Campagna R., Offidani A. (2018). Nicotinamide N-methyltransferase: Potential involvement in cutaneous malignant melanoma. Melanoma. Res..

[114] Bacchetti T., Salvolini E., Pompei V., Campagna R., Molinelli E., Brisigotti V., Togni L., Lucarini G., Sartini D., Campanati A. (2021). Paraoxonase-2: A potential biomarker for skin cancer aggressiveness. Eur. J. Clin. Investig..

[115] Campagna R., Pozzi V., Sartini D., Salvolini E., Brisigotti V., Molinelli E., Campanati A., Offidani A., Emanuelli M. (2021). Beyond Nicotinamide Metabolism: Potential Role of Nicotinamide N-Methyltransferase as a Biomarker in Skin Cancers. Cancers.

[116] Campagna R., Bacchetti T., Salvolini E., Pozzi V., Molinelli E., Brisigotti V., Sartini D., Campanati A., Ferretti G., Offidani A. (2020). Paraoxonase-2 Silencing Enhances Sensitivity of A375 Melanoma Cells to Treatment with Cisplatin. Antioxidants.

[117] Huo Y., Gao Y., Ruan J., Wang L., Li H., Hong G. (2025). A qualitative prognostic biomarker for melanoma based on the relative methylation orderings of CpG loci. Epigenetics.

[118] Weide B., Elsässer M., Büttner P., Pflugfelder A., Leiter U., Eigentler T.K., Bauer J., Witte M., Meier F., Garbe C. (2012). Serum markers lactate dehydrogenase and S100B predict independently disease outcome in melanoma patients with distant metastasis. Br. J. Cancer.

[119] Zhuang L., Scolyer R.A., Murali R., McCarthy S.W., Zhang X.D., Thompson J.F., Hersey P. (2010). Lactate dehydrogenase 5 expression in melanoma increases with disease progression and is associated with expression of Bcl-XL and Mcl-1, but not Bcl-2 proteins. Mod. Pathol..

[120] Lin J., Yang Q., Wilder P.T., Carrier F., Weber D.J. (2010). The Calcium-binding Protein S100B Down-regulates p53 and Apoptosis in Malignant Melanoma. J. Biol. Chem..

[121] Deckers E.A., Kruijff S., Brouwers A.H., van der Steen K., Hoekstra H.J., Thompson J.F., Vállez García D., Wevers K.P. (2020). The association between active tumor volume, total lesion glycolysis and levels of S-100B and LDH in stage IV melanoma patients. Eur. J. Surg. Oncol..

[122] Syeda M.M., Wiggins J.M., Corless B.C., Long G.V., Flaherty K.T., Schadendorf D., Nathan P.D., Robert C., Ribas A., Davies M.A. (2021). Circulating tumour DNA in patients with advanced melanoma treated with dabrafenib or dabrafenib plus trametinib: A clinical validation study. Lancet Oncol..

[123] Wouters J., Vizoso M., Martinez-Cardus A., Carmona F.J., Govaere O., Laguna T., Joseph J., Dynoodt P., Aura C., Foth M. (2017). Comprehensive DNA methylation study identifies novel progression-related and prognostic markers for cutaneous melanoma. BMC Med..

[124] Dankort D., Curley D.P., Cartlidge R.A., Nelson B., Karnezis A.N., Damsky W.E., You M.J., DePinho R.A., McMahon M., Bosenberg M. (2009). BrafV600E cooperates with Pten loss to induce metastatic melanoma. Nat. Genet..

[125] Huang F.W., Hodis E., Xu M.J., Kryukov G.V., Chin L., Garraway L.A. (2013). Highly Recurrent *TERT* Promoter Mutations in Human Melanoma. Science.

[126] Krauthammer M., Kong Y., Bacchiocchi A., Evans P., Pornputtapong N., Wu C., McCusker J.P., Ma S., Cheng E., Straub R. (2015). Exome sequencing identifies recurrent mutations in NF1 and RASopathy genes in sun-exposed melanomas. Nat. Genet..

[127] Mocellin S., Zavagno G., Nitti D. (2008). The prognostic value of serum S100B in patients with cutaneous melanoma: A meta-analysis. Int. J. Cancer.

[128] Feng S.N., Cen X.T., Tan R., Wei S.S., Sun L.D. (2021). The prognostic value of circulating tumor DNA in patients with melanoma: A systematic review and meta-analysis. Transl. Oncol..

[129] Ascierto P.A., Dummer R., Gaudy-Marqueste C., Bowyer S., Lipson E.J., Ghisoni E., Middleton M.R., Ratto B., Jackson W.J., Cheong A. (2024). Efficacy and safety of triplet nivolumab, relatlimab, and ipilimumab (NIVO + RELA + IPI) in advanced melanoma: Results from RELATIVITY-048. J. Clin. Oncol..

[130] Long G.V., Shklovskaya E., Satgunaseelan L., Mao Y., da Silva I.P., Perry K.A., Diefenbach R.J., Gide T.N., Shivalingam B., Buckland M.E. (2025). Neoadjuvant triplet immune checkpoint blockade in newly diagnosed glioblastoma. Nat. Med..

[131] Ribas A., Eroglu Z., Perez J.M.M.T., Pace B.D., Wang T., Ghosh S., Dhar A., Borgovan T., Waszak A., Davar D. (2022). AMBER parts 1c and 1e: A phase 1 study of cobolimab plus dostarlimab in patients (pts) with advanced/metastatic melanoma. J. Clin. Oncol..

[132] Mooradian M., Karunamurthy A., Wang H., Buchbinder E.I., Rapisuwon S., Cohen J.V., Gibney G.T., Sullivan R.J., Luke J.J., Najjar Y.G. (2025). Randomized phase II study of neoadjuvant (neoadj) anti-PD-1 dostarlimab (D) vs. D + anti-TIM-3 cobolimab (C) in high-risk resectable melanoma (mel) (NEO-MEL-T): Primary analysis. J. Clin. Oncol..

[133] Zhang Q., Bi J., Zheng X., Chen Y., Wang H., Wu W., Wang Z., Wu Q., Peng H., Wei H. (2018). Blockade of the checkpoint receptor TIGIT prevents NK cell exhaustion and elicits potent anti-tumor immunity. Nat. Immunol..

[134] Yu X., Harden K., Gonzalez L.C., Francesco M., Chiang E., Irving B., Tom I., Ivelja S., Refino C.J., Clark H. (2009). The surface protein TIGIT suppresses T cell activation by promoting the generation of mature immunoregulatory dendritic cells. Nat. Immunol..

[135] Boles K.S., Vermi W., Facchetti F., Fuchs A., Wilson T.J., Diacovo T.G., Cella M., Colonna M. (2009). A novel molecular interaction for the adhesion of follicular CD4 T cells to follicular DC. Eur. J. Immunol..

[136] Dummer R., Robert C., Scolyer R.A., Taube J.M., Tetzlaff M.T., Menzies A.M., Hill A., Grob J.J., Portnoy D.C., Lebbe C. (2025). Neoadjuvant anti-PD-1 alone or in combination with anti-TIGIT or an oncolytic virus in resectable stage IIIB-D melanoma: A phase 1/2 trial. Nat. Med..

[137] Pietrobono S., Morandi A., Gagliardi S., Gerlini G., Borgognoni L., Chiarugi P., Arbiser J.L., Stecca B. (2016). Down-Regulation of SOX2 Underlies the Inhibitory Effects of the Triphenylmethane Gentian Violet on Melanoma Cell Self-Renewal and Survival. J. Investig. Dermatol..

[138] Brown C.E., Hibbard J.C., Alizadeh D., Blanchard M.S., Natri H.M., Wang D., Ostberg J.R., Aguilar B., Wagner J.R., Paul J.A. (2024). Locoregional delivery of IL-13Rα2-targeting CAR-T cells in recurrent high-grade glioma: A phase 1 trial. Nat. Med..

[139] Shah P.D., Huang A.C., Xu X., Orlowski R., Amaravadi R.K., Schuchter L.M., Zhang P., Tchou J., Matlawski T., Cervini A. (2023). Phase I Trial of Autologous RNA-electroporated cMET-directed CAR T Cells Administered Intravenously in Patients with Melanoma and Breast Carcinoma. Cancer Res. Commun..

[140] Gargett T., Truong N.T.H., Gardam B., Yu W., Ebert L.M., Johnson A., Yeo E.C.F., Wittwer N.L., Tapia Rico G., Logan J. (2024). Safety and biological outcomes following a phase 1 trial of GD2-specific CAR-T cells in patients with GD2-positive metastatic melanoma and other solid cancers. J. Immunother. Cancer.

[141] Lei W., Liu H., Deng W., Chen W., Liang Y., Gao W., Yuan X., Guo S., Li P., Wang J. (2025). Safety and feasibility of 4-1BB co-stimulated CD19-specific CAR-NK cell therapy in refractory/relapsed large B cell lymphoma: A phase 1 trial. Nat. Cancer.

[142] Grote S., Ureña-Bailén G., Chan K.C., Baden C., Mezger M., Handgretinger R., Schleicher S. (2021). In Vitro Evaluation of CD276-CAR NK-92 Functionality, Migration and Invasion Potential in the Presence of Immune Inhibitory Factors of the Tumor Microenvironment. Cells.

[143] Seery T.E., Nangia C.S., McKean H.A., Sender L.S., Bhar P., Reddy S.K., Soon-Shiong P. (2022). Promising survival and disease control in third-line or greater metastatic or locally advanced pancreatic cancer patients following chemo-radiation and novel combination of aldoxorubicin, N-803 IL-15 superagonist, and PDL1- NK cell therapy. J. Clin. Oncol..

[144] He G., Li Y., Zeng Y., Zhang Y., Jiang Q., Zhang Q., Zhu J., Gong J. (2024). Advancements in melanoma immunotherapy: The emergence of Extracellular Vesicle Vaccines. Cell Death Discov..

[145] Chen G., Huang A.C., Zhang W., Zhang G., Wu M., Xu W., Yu Z., Yang J., Wang B., Sun H. (2018). Exosomal PD-L1 contributes to immunosuppression and is associated with anti-PD-1 response. Nature.

[146] Shu S., Matsuzaki J., Want M.Y., Conway A., Benjamin-Davalos S., Allen C.L., Marina K., Sebastiano B., Adekunle O., Hans M. (2020). An Immunosuppressive Effect of Melanoma-derived Exosomes on NY-ESO-1 Antigen-specific Human CD8+ T Cells is Dependent on IL-10 and Independent of BRAFV600E Mutation in Melanoma Cell Lines. Immunol. Investig..

[147] Guo D., Jain R., Hwang J.S., Weninger W., Beaumont K.A., Tikoo S. (2020). RAB27A/Melanophilin Blocker Inhibits Melanoma Cell Motility and Invasion. J. Investig. Dermatol..

[148] Guo D., Lui G.Y.L., Lai S.L., Wilmott J.S., Tikoo S., Jackett L.A., Quek C., Brown D.L., Sharp D.M., Kwan R.Y.Q. (2019). RAB27A promotes melanoma cell invasion and metastasis via regulation of pro-invasive exosomes. Int. J. Cancer.

[149] Guo D., Beaumont K.A., Sharp D.M., Lui G.Y.L., Weninger W., Haass N.K., Tikoo S. (2020). Abrogation of RAB27A expression transiently affects melanoma cell proliferation. Pigment Cell Melanoma Res..

[150] Babaei S., Fadaee M., Abbasi-kenarsari H., Shanehbandi D., Kazemi T. (2024). Exosome-based immunotherapy as an innovative therapeutic approach in melanoma. Cell Commun. Signal..

[151] Alia Moosavian S., Hashemi M., Etemad L., Daneshmand S., Salmasi Z. (2022). Melanoma-derived exosomes: Versatile extracellular vesicles for diagnosis, metastasis, immune modulation, and treatment of melanoma. Int. Immunopharmacol..

[152] Escudier B., Dorval T., Chaput N., André F., Caby M.-P., Novault S., Flament C., Leboulaire C., Borg C., Amigorena S. (2005). Vaccination of metastatic melanoma patients with autologous dendritic cell (DC) derived-exosomes: Results of thefirst phase I clinical trial. J. Transl. Med..

[153] Bekes M., Langley D.R., Crews C.M. (2022). PROTAC targeted protein degraders: The past is prologue. Nat. Rev. Drug Discov..

[154] Gough S.M., Flanagan J.J., Teh J., Andreoli M., Rousseau E., Pannone M., Bookbinder M., Willard R., Davenport K., Bortolon E. (2024). Oral Estrogen Receptor PROTAC Vepdegestrant (ARV-471) Is Highly Efficacious as Monotherapy and in Combination with CDK4/6 or PI3K/mTOR Pathway Inhibitors in Preclinical ER+ Breast Cancer Models. Clin. Cancer Res..

[155] Alhassan S.O., Abd Elmageed Z.Y., Errami Y., Wang G., Abi-Rached J.A., Kandil E., Zerfaoui M. (2025). BRAFV600E-PROTAC versus inhibitors in melanoma cells: Deep transcriptomic characterisation. Clin. Transl. Med..

[156] Posternak G., Tang X., Maisonneuve P., Jin T., Lavoie H., Daou S., Orlicky S., Goullet de Rugy T., Caldwell L., Chan K. (2020). Functional characterization of a PROTAC directed against BRAF mutant V600E. Nat. Chem. Biol..

[157] Ohoka N., Suzuki M., Uchida T., Tsukumo Y., Yoshida M., Inoue T., Ohki H., Naito M. (2022). Development of a potent small-molecule degrader against oncogenic BRAFV600E protein that evades paradoxical MAPK activation. Cancer Sci..

[158] Alabi S., Jaime-Figueroa S., Yao Z., Gao Y., Hines J., Samarasinghe K.T.G., Vogt L., Rosen N., Crews C.M. (2021). Mutant-selective degradation by BRAF-targeting PROTACs. Nat. Commun..

[159] Young R.J., Waldeck K., Martin C., Foo J.H., Cameron D.P., Kirby L., Do H., Mitchell C., Cullinane C., Liu W. (2014). Loss of CDKN2A expression is a frequent event in primary invasive melanoma and correlates with sensitivity to the CDK4/6 inhibitor PD0332991 in melanoma cell lines. Pigment Cell Melanoma Res..

[160] Zaemes J., Ali A., Kathryn V., Atkins M. (2020). A Patient with Melanoma that Became Sensitized to Immunotherapy After Treatment with a CDK4/6 Inhibitor. Immunotherapy.

[161] Goel S., DeCristo M.J., Watt A.C., BrinJones H., Sceneay J., Li B.B., Khan N., Ubellacker J.M., Xie S., Metzger-Filho O. (2017). CDK4/6 inhibition triggers anti-tumour immunity. Nature.

[162] Chehelgerdi M., Chehelgerdi M., Allela O.Q.B., Pecho R.D.C., Jayasankar N., Rao D.P., Thamaraikani T., Vasanthan M., Viktor P., Lakshmaiya N. (2023). Progressing nanotechnology to improve targeted cancer treatment: Overcoming hurdles in its clinical implementation. Mol. Cancer.

[163] Bombelli F.B., Webster C.A., Moncrieff M., Sherwood V. (2014). The scope of nanoparticle therapies for future metastatic melanoma treatment. Lancet Oncol..

